# Effects of physical activity interventions on anthropometric indicators and health indices in Chilean children and adolescents: A protocol for systematic review and/or meta-analysis

**DOI:** 10.1097/MD.0000000000033894

**Published:** 2023-05-26

**Authors:** Andrés Godoy-Cumillaf, Claudio Farías-Valenzuela, Daniel Duclos-Bastías, Frano Giakoni-Ramírez, Jaime Vásquez-Gómez, José Bruneau-Chávez, Bruno Bizzozero-Peroni

**Affiliations:** a Universidad Autónoma de Chile, Chile, Grupo de investigación en Educación Física, Salud y Calidad de Vida, Pedagogía en Educación Física, Temuco, Chile; b Instituto del Deporte, Universidad de las Américas, Santiago, Chile; c Escuela de Educación Física, Pontificia Universidad Católica de Valparaíso, Valparaíso, Chile; d Faculty of Education and Social Sciences, Universidad Andres Bello, Las Condes, Chile; e Centro de Investigación de Estudios Avanzados del Maule (CIEAM), Universidad Católica del Maule, Talca, Chile; f Laboratorio de Rendimiento Humano, Universidad Católica del Maule, Talca, Chile; g Departamento de Educación Física, Deportes y Recreación, Universidad de la Frontera, Temuco, Chile; h Health and Social Research Center, Universidad de Castilla-La Mancha, Cuenca, Spain; i Instituto Superior de Educación Física, Universidad de la República, Rivera, Uruguay.

**Keywords:** anthropometric, Chile, health, systematic review

## Abstract

Physical activity interventions have been a proven effective means of preventing or treating overweight and obesity in children and adolescents. The results of these interventions in many cases are based on the determination of the effect they produce on anthropometric evaluations, which allow the calculation of health indices. However, the effects of physical activity interventions on anthropometric assessments in Chilean children and adolescents have not been systematized. The objective of this study is to provide a detailed protocol for a systematic review with meta-analysis that synthesizes the available evidence on the effect of physical activity interventions on anthropometric indicators and health indices in Chilean children and adolescents and identifies the field-based methods and health indices most used for body composition estimation.

**Methods:**

This protocol was performed according to the PRISMA declaration. MEDLINE (PubMed), Web of Science, Scopus, and Scielo databases will be systematically searched. Eligible studies will include randomized controlled trials (RCTs), non-RCTs and pre-post studies.

**Conclusion:**

This systematic review and meta-analysis protocol is designed to provide up-to-date evidence that could significantly assist public health policy makers and implementers of physical activity interventions through evidence-based guidance and recommendations.

## 1. Introduction

Overweight and obesity in Latin America children and adolescents has increased considerably in recent years.^[1]^ Regarding Chile, 27% overweight and 31% obesity were estimated in this population in 2021,^[2]^ indicating an alarming public health situation because they are important risk factors for hypertension,^[3]^ diabetes mellitus,^[4]^ cardiovascular diseases,^[5]^ some types of cancer,^[6]^ and premature death.^[7]^

Physical activity (PA) interventions have been a proven effective means to prevent or treat overweight and obesity and thus improve health-related values in children and adolescents.^[8–10]^ The results of these interventions in many cases are based on the determination of the effect they produce on anthropometry indicators (e.g., weight, height, body mass index – BMI) that are repeatedly used to estimate body fat distribution.^[11,12]^ In turn, it is common for PA interventions to assess other field-based methods for body composition estimation, such as skinfolds or body circumferences.^[13,14]^ Consequently, these anthropometry indicators allow the calculation of health indices, such as height-for-age or waist-to-hip ratio. Although technological advances have allowed the creation of more precise instruments such as magnetic resonance imaging or dual-energy X-ray absorptiometry,^[15,16]^ anthropometric measures and health indices are widely used, mainly due to their low cost, simple application, and validity for estimating body size.^[17,18]^

In this context, government authorities in Chile have developed manuals that incorporate anthropometric indicators and the calculation of health indices to monitor the comprehensive health of children and adolescents.^[19–21]^ Specifically, they are included for the diagnosis and follow-up of PA interventions aimed at preventing or treating overweight and obesity. However, the effects of PA interventions on the different types of anthropometric indicators used are not reported in these guidelines.

Previous studies support the beneficial effect of PA interventions in improving anthropometric outcomes in children and adolescents.^[8–10]^ Latin American systematic reviews conclude that PA interventions can be effective in achieving improvements in anthropometric indicators.^[22–25]^ However, the only meta-analysis performed for Latin American children and adolescents concluded that PA interventions did not report significant beneficial effects on BMI and did not examine results for other anthropometric indicators or health indices.^[26–28]^ Specifically, on anthropometric indicators and health indices in Chilean children and adolescents, the effects of PA interventions have not been systematized. In addition, the most used anthropometric variables and health indices have not been summarized, which could lead to differences in the choice of measures to be used and, therefore, generate difficulties in the comparison and extrapolation of anthropometric results for Chilean children and adolescents. For this reason, knowledge of the most used anthropometric indicators is increasingly valuable, as they allow a double opportunity: first, to estimate the body fat distribution and predict the health status of an individual, and second, to guide national public health policies for children and adolescents through evidence-based recommendations.

## 2. Objective

The objective of this study is to provide a detailed a systematic review and meta-analysis protocol that synthesizes the existing evidence on the effect of PA interventions on anthropometric indicators and health indices in Chilean children and adolescents and identifies the anthropometric methods and health indices most used for body composition estimation.

## 3. Methods

The procedure was carried out in agreement with the Preferred Reporting Items for Systematic Reviews and Meta-Analyses Protocols (PRISMA-P) statement,^[29]^ available in Appendix 1 (Table S1, Supplemental Digital Content, http://links.lww.com/MD/J36). The PRISMA 2020 items^[30]^ and the Cochrane Collaboration Handbook for Systematic Reviews of Interventions^[31]^ will be used to carry out the systematic review and meta-analysis. The entirety of the current protocol’s content has been registered in PROSPERO (CRD42023398677).

### 3.1. Eligibility criteria

The patient, intervention, comparison, outcome, and study design framework^[31]^ was used to establish inclusion and exclusion criteria. The studies should meet the next criteria: participants: healthy children and adolescents (i.e., without any reported disease) between 4 and 18 years old living in Chile; intervention: PA programs (e.g., exercise, sports, games, dancing); comparison: control groups as non-PA interventions; outcome: anthropometric indicators (e.g., weight, height, waist circumference) or health indices (e.g., body mass index, waist-hip ratio); and study design: randomized controlled trials (RCTs), non-RCTs, or pre-post studies. In the meantime, the following exclusion criteria will be established: qualitative studies and studies that reproduce data published in another study.

Studies that perform PA intervention supplemented with other activities (e.g., lifestyle interventions such as diet or sleep) will be included in the review.

### 3.2. Search strategy

Bibliographic searches will be conducted using various electronic databases including MEDLINE (via PubMed), Scopus, Web of Science, and Scielo. The search will cover the period from the beginning of the database until April 20, 2023. The search will be restricted to keywords, titles, and abstracts, and search terms will be combined using the Boolean operator OR and simultaneously searched with other search groups based on the patient, intervention, comparison, outcome, and study design elements (population, intervention, comparison, outcome, and study design) using the Boolean operator AND (Table 1). To find root words, proximity operators (“*”) will be utilized.

**Table 1:** Search strategy or the MEDLINE database.

PICO components	Keywords
#1 Populations	child* OR boys OR girls OR teen OR pediatric OR adolescents OR youth OR young OR chile*
#2 Intervention	“physical activity” OR exercise OR training OR “prevention program” OR sports
#3 Outcome	“health indices” OR weight OR height OR “waist circumference” OR “hip circumference” OR “neck circumference” OR skinfold OR “body mass index” OR BMI OR “body composition” OR “body measures” OR “body size” OR anthropometr* OR “waist-hip ratio” OR “height waist ratio”
#4 Study design	“randomized controlled trial” OR RCT OR “non-randomized clinical trial” OR “pre-post study” OR “controlled trial” OR “clinical trial” OR intervention OR randomi*
Search strategy	#1 AND #2 AND #3 AND #4

Proximity operators (*) will be used to search for root words.

### 3.3. Selection of studies

All references obtained from the database will be introduced into Mendeley Reference Manager (V.1.19.8; Elsevier, London, UK). After removing duplicates, 2 researchers will independently examine the titles and abstracts of the retrieved articles to determine which studies are eligible for the systematic review. Abstracts that meet the inclusion criteria or provide insufficient information on the inclusion/exclusion criteria will be further evaluated through full-text reading. The researchers will then assess both included and excluded studies to confirm the reasoning behind each decision. If there are discrepancies between the researchers regarding selection, a third investigator will resolve them and make the final decision based solely on the inclusion/exclusion criteria.

### 3.4. Data collection process

**Figure 1.**
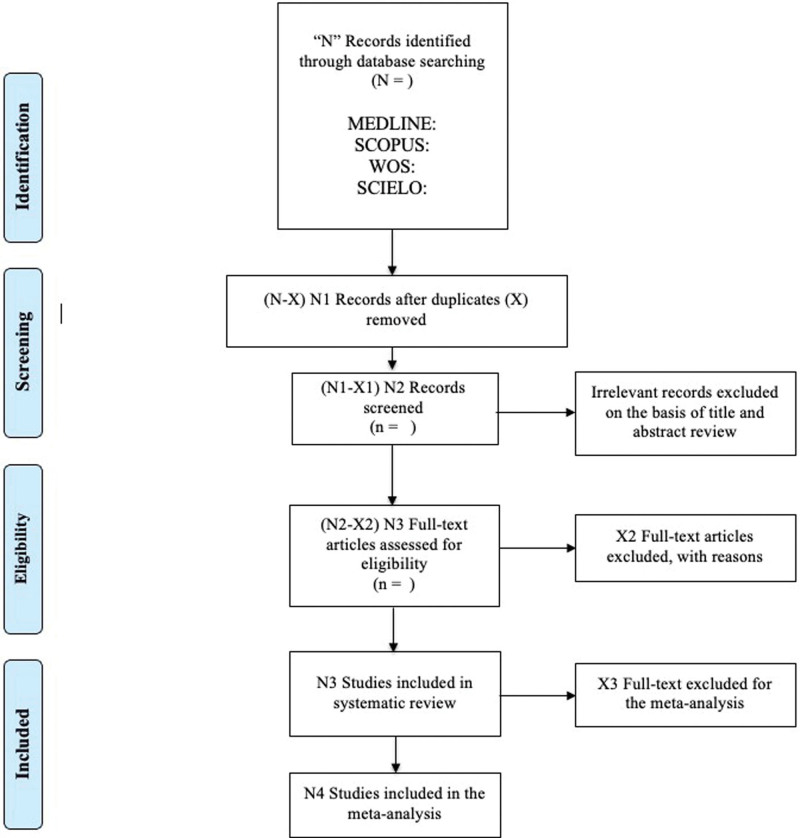
PRISMA flow diagram.

The data collection procedure will follow the PRISMA flowchart (Fig. 1). One investigator will extract descriptive data into a spreadsheet using a standardized study-specific template, and a second investigator will verify its accuracy. If necessary, additional information will be requested from the corresponding authors by e-mail. The following information will be extracted from the full texts of the identified studies: name of first author and year of publication; study design; ages of participants; number of participants; type of population; duration and characteristics of the intervention; control and intervention group characteristics; anthropometric indicators used; health indices used; references used for calculation of indices; and pre-post results on anthropometric indicators and health indices.

### 3.5. Bias risk assessment

Two reviewers will independently evaluate the risk of bias, using the guidelines from the Cochrane Collaboration Manual. In case of any discrepancy, the reviewers will hold a discussion to reach a consensus. However, if a consensus cannot be reached, a third reviewer will intervene to settle the disagreement. The level of agreement between reviewers will be reported by calculating kappa statistics.^[32]^

The Cochrane Collaboration tool (RoB 2.0)^[33]^ will be utilized to assess the risk of bias of RCTs. Non-RCTs and quasi-experimental studies will be evaluated using the Risk of Bias In Non-randomized Studies of Interventions tool.^[34]^

### 3.6. Data synthesis

The researchers will summarize the key characteristics of each study included in the analysis, including the general study profile, methods, participant characteristics, and results (Table 2). For intervention studies reporting pre- and post-intervention anthropometric indicators and/or health indices, a meta-analysis will be conducted. Studies with insufficient data for meta-analysis will only be included in the systematic review. The pooled mean differences with 95% confidence intervals will be combined using Stata/SE software (Version 15.0;185 StataCorp, College Station, TX) if a meta-analysis is appropriate. A fixed effects model will be used if there is no evidence of heterogeneity; otherwise, a random effects model will be used. The degree of study heterogeneity will be assessed using the *I*^2^ statistic. The corresponding *P* values will also be considered.^[31]^

**Table 2:** Characteristics of studies included in the systematic review and meta-analysis.

Reference	Study design	Age distribution	Sample size	Type of population	Physical-activity intervention	Group characteristics	Anthropometric indicators	Health indices	References	Results anthropometric indicators	Results health indices
Author information and year of publication	Design of the study	Age (years) of the participants range or means ± SD	Number of participants	Population characteristics (low weight, normal weight, overweight, obese)	Duration and characteristics of the intervention	Control and intervention group characteristics	Anthropometric indicators used	Health indices used	References used for calculation of health indices	Pre-post results	Pre-post results

In the case of RCTs, the data obtained from intention-to-treat analyses will be considered, if available. Two analyses will be conducted: mean difference in anthropometric indicators and/or health indices before and after the PA-based intervention compared to a control group and mean difference in anthropometric indicators and/or health indices before and after the intervention based on PA, without a control group. Moreover, a funnel plot will be used to evaluate publication bias, following the Egger method.^[35]^

If available, subgroup analyses and meta-regression will be conducted to investigate potential sources of heterogeneity, such as sex (male or female), age (children aged 4–12 years or adolescents aged 12–18 years), population (general, indigenous, or mixed), study design (RCTs, non-RCTs, or pre-post studies), BMI (underweight, normal weight, overweight, or obesity), intervention type (PA-based programs only or combined with other lifestyle interventions), type of PA (e.g., exercise, sports, games, dancing), intervention duration (above or below 12 weeks), intervention setting (e.g., school, outdoor, clinic), and weekly duration of PA programs (above or below 150 minutes). Furthermore, the methodological quality of the included studies will be considered for additional subgroup analyses. Additionally, sensitivity analyses will be performed by systematically removing individual studies from the pooled analyses to determine the robustness of the summary estimates and whether any single study accounts for a significant proportion of heterogeneity.

### 3.7. Ethics and dissemination

Since in this systematic review protocol the investigators will not collect primary data, ethics committee approval and/or informed consent from patients will not be needed.

We will publish a scientific paper in an open access peer-reviewed and indexed journal. Additionally, the authors will attend and present findings from this systematic review at both national and international scientific conferences. We will engage relevant stakeholders, including decision makers, schools, pediatrics, and researchers, through the dissemination of symposiums, infographics, and videos.

## 4. Discussion

The aim of this protocol is to describe a standardized methodology to systematically review the available scientific literature on the effect of PA interventions on anthropometric indicators and health indices in Chilean children and adolescents. Moreover, the most used measures of body composition (i.e., field-based methods and health indices) in this population will be synthesized.

Although anthropometric measurements are used to determine the effect of PA interventions on body composition,^[11–14]^ to our knowledge, there are no systematic reviews with meta-analyses that have answered the following questions in Chilean children and adolescents: What are the effects of PA interventions on anthropometry assessments? What are the most widely used field-based methods and health indices methods to measure the effect of PA-based strategies? Finally, is it possible to compare the values obtained in anthropometric indicators and health indices of Chilean children and adolescents with their age- and sex-matched peers in other parts of the world?

As data on obesity grow, clinicians and researchers are increasingly interested in assessing body composition to better manage it, improve prevention and therapeutic decisions, and optimize health outcomes. In this context, anthropometric measurements are noninvasive, inexpensive, and suitable methods to assess the health status of young people. This study protocol describes a standardized methodology for the development of a systematic review that will allow us to answer the above research questions, establishing a comprehensive picture of the available scientific evidence on the effects of PA interventions on anthropometric variables and health indices in children and adolescents in Chile. Therefore, the findings of our study could contribute to the formation of public health policies at the national level to reduce the high levels of overweight and obesity in the child and adolescent population of Chile.

Among the possible limitations of this systematic review and meta-analysis, the inherent limitations of this type of study (e.g., publication bias, reporting bias, poor statistical analysis, low methodological quality, and inadequate reporting of methods and findings) should be anticipated. In addition, we hypothesized considerable heterogeneity in the meta-analysis due to potential variability in PA intervention programs and anthropometric outcomes.

## 5. Conclusion

The lack of homogenized information on anthropometric indicators and health indices used to determine the effect of physical activity interventions applied in children and adolescents in Chile highlights the need to systematically review the available scientific literature. Such information could significantly help public health policy makers and those responsible for the implementation of physical activity interventions through evidence-based guidelines and recommendations. In addition, it may be useful to identify knowledge gaps in the scientific literature to guide future studies.

## Acknowledgments

This publication is part of the research project of Andrés Godoy Cumillaf, and has been financed by the Universidad Autónoma de Chile, through the internal project DIP 249-2022 of the Vicerrectoría de Investigación y Doctorados.

## Author contributions

**Conceptualization:** Andrés Godoy-Cumillaf.

**Data curation:** Bruno Bizzozero-Peroni.

**Formal analysis:** Jaime Vásquez-Gómez, José Bruneau-Chávez.

**Funding acquisition:** Andrés Godoy-Cumillaf.

**Methodology:** Daniel Duclos-Bastías, Frano Giakoni-Ramírez.

**Supervision:** Daniel Duclos-Bastías, Frano Giakoni-Ramírez, José Bruneau-Chávez.

**Validation:** Claudio Farías-Valenzuela, Frano Giakoni-Ramírez, Jaime Vásquez-Gómez.

**Writing – original draft:** Andrés Godoy-Cumillaf, José Bruneau-Chávez, Bruno Bizzozero-Peroni.

**Writing – review & editing:** Claudio Farías-Valenzuela, Daniel Duclos-Bastías, Frano Giakoni-Ramírez, Jaime Vásquez-Gómez.

## Supplementary Material
